# Predicting functional consequences of mutations using molecular interaction network features

**DOI:** 10.1007/s00439-021-02329-5

**Published:** 2021-08-25

**Authors:** Kivilcim Ozturk, Hannah Carter

**Affiliations:** 1 Division of Medical Genetics, Department of Medicine, University of California San Diego, La Jolla, CA USA; 2 Bioinformatics and Systems Biology Program, University of California San Diego, La Jolla, CA USA; 3 Moores Cancer Center, University of California San Diego, La Jolla, CA USA

## Abstract

Variant interpretation remains a central challenge for precision medicine. Missense variants are particularly difficult to understand as they change only a single amino acid in a protein sequence yet can have large and varied effects on protein activity. Numerous tools have been developed to identify missense variants with putative disease consequences from protein sequence and structure. However, biological function arises through higher order interactions among proteins and molecules within cells. We therefore sought to capture information about the potential of missense mutations to perturb protein interaction networks by integrating protein structure and interaction data. We developed 16 network-based annotations for missense mutations that provide orthogonal information to features classically used to prioritize variants. We then evaluated them in the context of a proven machine-learning framework for variant effect prediction across multiple benchmark datasets to demonstrate their potential to improve variant classification. Interestingly, network features resulted in larger performance gains for classifying somatic mutations than for germline variants, possibly due to different constraints on what mutations are tolerated at the cellular versus organismal level. Our results suggest that modeling variant potential to perturb context-specific interactome networks is a fruitful strategy to advance in silico variant effect prediction.

**Supplementary Information:**

The online version contains supplementary material available at 10.1007/s00439-021-02329-5.

## Introduction

Advances in high-throughput sequencing technologies have resulted in the rapid accumulation of genomic data and allowed profiling of patient genomes in clinical settings. Such studies frequently uncover previously unobserved and uncharacterized genetic variants of ambiguous relevance to health, making variant interpretation an important challenge in precision medicine (Fernald et al. 2011). Missense mutations are particularly challenging as they only change a single amino acid in a protein sequence yet can have effects spanning no difference to complete loss of function. Numerous methods have been developed to prioritize functional missense variants (Adzhubei et al. 2010; Cooper and Shendure 2011; Hecht et al. 2015; Ioannidis et al. 2016; Kircher et al. 2014; Liu et al. 2020; Ng and Henikoff 2003; Pejaver et al. 2020; Ponzoni et al. 2020). Typically, these tools rely on protein sequence/structure information to predict variant effects at the protein level, and the scores they provide tend to capture coarse grained estimates of impact (e.g., damaging, benign, and tolerated).

Biological functions and cellular behaviors arise from interactions among proteins and other molecules within cells, and biological systems evolve to be robust to random error (Félix and Barkoulas 2015). Diseases are often associated with perturbations to protein interactions, and different perturbations can result in different phenotypes (Vidal et al. 2011), and the level of impact caused by mutations to the underlying molecular interaction network may determine the likelihood of generating a phenotype (Capriotti et al. 2019). For example, loss-of-function mutations were more likely to be tolerated when they affected proteins at the periphery of the interactome (Khurana et al. 2013). Similarly, variants that otherwise were predicted to have little effect were more likely to be deleterious if they had a large number of interaction partners (Yates et al. 2014) and de novo missense variants in autism probands with functional Polyphen2 predictions were enriched at protein interfaces of more central proteins relative to similar mutations in control siblings (Chen et al. 2018). Thus, a protein’s location within the system provides biological context that may be important for understanding the effects of mutations (Ozturk et al. 2018).

Within proteins, different mutations may have different effects on protein functions (Sahni et al. 2013; Zhong et al. 2009). While destabilizing mutations at the core of a protein are likely to interfere with all protein activities, mutations on the surface could potentially interfere with specific protein activities while preserving others (Zhong et al. 2009). In this way, different mutations targeting the same protein might perturb its interactions differently, affecting different pathways that the protein is involved in, and resulting in different disease phenotypes (Engin et al. 2015). Indeed, analyses have demonstrated an unexpected enrichment of Mendelian mutations (David et al. 2012; Guo et al. 2013; Wang et al. 2012) and somatic mutations (Engin et al. 2016; Kamburov et al. 2015; Porta-Pardo et al. 2015; Raimondi et al. 2016) at protein interaction interfaces. Although protein structure-derived features have long been integral to variant classification, some more recent features capturing 3D location of mutations within key protein regions including local density of mutation and location at interface regions have emerged (Iqbal et al. 2020; Laskowski et al. 2020; Tokheim et al. 2016; Tokheim and Karchin 2019). While these features begin to capture information about the potential variants to affect distinct interactions, they do not incorporate context about the importance of specific interactions within the larger interactome.

**Fig. 1:**
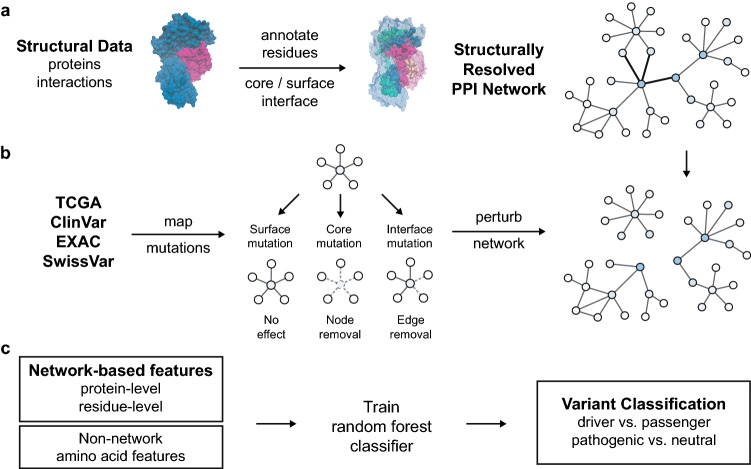
Overview of the method. **a** Constructing a structurally resolved PPI network. **b** Mapping mutations to perturbed network architectures. **c** Designing protein-level and residue-level network-based features and using a machine-learning framework to evaluate their potential for variant classification alone and in combination with classic non-network amino acid features

Based on the above, we sought to assess the potential for artificial intelligence-based methods for variant interpretation to derive new information from molecular interaction data. We first integrated structure and protein–protein interaction (PPI) networks to enable systematic annotation of proteins according to location and interactions (Fig. 1a). We mapped various germline variants and somatic mutations to network edges to describe their potential to impact biological function (Fig. 1b). We then designed features capturing information about proteins and amino acids in the context of their importance to the network architecture and evaluated them within a machine-learning variant classification framework (Fig. 1c). We found that network-based features capture orthogonal information to classical amino acid (AA) sequence/structure-based features and can improve variant classification, though they may be more informative for some variant classification tasks than others.

## Results

### Disease-causing genes are central in PPI networks

**Fig. 2:**
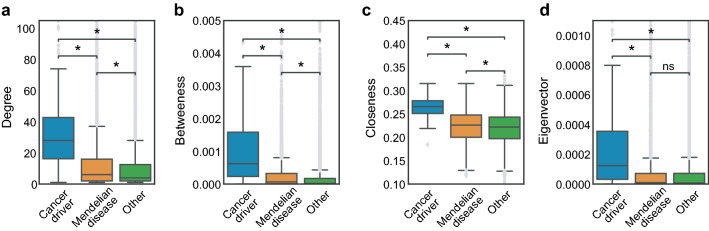
Disease genes are central in PPI networks. Boxplots showing distributions of **a** degree, **b** betweenness, **c** closeness, and **d** eigenvector centralities of cancer driver, Mendelian disease, and other genes (Mann–Whitney *U* test with Bonferroni correction; *p < 1e−04)

The architectures of biological networks can provide important information for understanding the pathogenesis of mutations (Barabási et al. 2011; Ozturk et al. 2018). The scale-free topology of PPI networks suggests that they are more tolerant to random failures, but variants affecting higher degree nodes are more likely to disrupt function (Albert et al. 2000). Indeed, when we compared disease genes using a high-confidence human PPI network of experimentally verified interactions from STRING (Szklarczyk et al. 2015), cancer driver (Vogelstein et al. 2013) and Mendelian disease genes (Amberger et al. 2015) score higher with various centrality measures than other genes (Fig. 2). This suggests that the network niche of a gene provides information about the potential of an amino acid substitution to create deleterious phenotypes, a relationship that has proven robust to study bias (Vinayagam et al. 2016); in our data, only node degree correlates weakly with the number of publications (Pearson *r* = 0.23, Fig. S1).

### Creating a structurally resolved PPI network

While disease mutations target proteins more central in interaction networks (Fig. 2), protein-level descriptors of centrality are not capable of distinguishing the effects of different mutations within proteins. Investigation of residue-specific network perturbations requires mapping of mutations to 3D protein structures and interaction interfaces, so that we can model their potential to affect network edges (Fig. 1b). We constructed a structurally resolved PPI network (called SRNet from here on) comprising 6230 proteins and 10,615 PPIs using 3D structures and homology models (Fig. 1a, Table S1). This network contains annotations for 530,668 interface residues, defined here as the subset of amino acid residues that mediate physical contact between proteins. Otherwise, amino acids are annotated according to location at the surface or core based on relative solvent accessible surface area calculated from protein 3D structures (Materials and methods). SRNet is an updated and extended version of our previous structurally resolved PPI network (Engin et al. 2016).

### Disease mutations frequently target interface or core residues

**Fig. 3:**
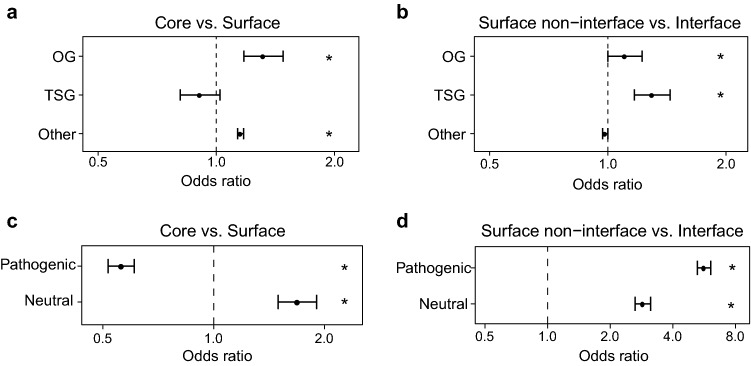
Analysis of structural location of missense disease mutations. Odds ratios (OR) and 95% confidence intervals using Fisher’s exact test are shown (*p < 0.05). Comparison of somatic mutations in oncogenes (OG), tumor suppressor genes (TSG), and other genes located at **a** core vs. surface residues, and **b** surface non-interface vs. surface interface residues. Comparison of pathogenic and neutral variants located at **c** core vs. surface residues, and **d** surface non-interface vs. surface interface residues. For **a** and **c**, an OR > 1 means that more mutations/variants were found at the surface. For **b** and **d,** an OR > 1 means more mutations/variants were found at interfaces

We further assessed the potential for SRNet to capture information about residue-based network-perturbation by analyzing location of mutations relative to core, surface, or interface regions. Similar to the finding in Engin et al. (2016), SRNet supports that somatic missense mutations in tumors (obtained from The Cancer Genome Atlas (TCGA) (Collins and Barker 2007)) target surface regions in oncogenes (OR 1.32, *p* = 1.4e−06) and other genes (OR 1.15, *p* = 1.07e−59), but are relatively depleted at surface regions in tumor suppressor genes (OR 0.91, *p* < 0.1) due to a larger proportion of core mutations (Fig. 3a), consistent with more loss-of-function mutations in tumor suppressors. However, when focusing only on surface positions, somatic mutations are more likely to be found at interface regions of oncogenes (OR 1.11, *p* < 0.05) and tumor suppressors (OR 1.30, *p* = 7.8e−07) relative to other genes (Fig. 3b). Analysis of pathogenic germline variants [ClinVar (Landrum et al. 2018)] versus neutral variants [EXAC (Lek et al. 2016), SwissVar (Mottaz et al. 2010), ClinVar (Landrum et al. 2018)] found similar trends. Pathogenic variants were relatively depleted at the surface (OR 0.56, *p* = 1.5e−42), suggesting that they were far more likely to affect core regions, whereas neutral variants were biased toward the surface (OR 1.69, *p* = 1e−19) (Fig. 3c). On protein surfaces, pathogenic variants were more often found at interface regions (OR 5.65, *p* = 2.2e−308), though neutral variants also showed increased odds of affecting an interface (OR 2.87, *p* = 2.6e−115) (Fig. 3d).

### Network-based features for variant classification

**Fig. 4:**
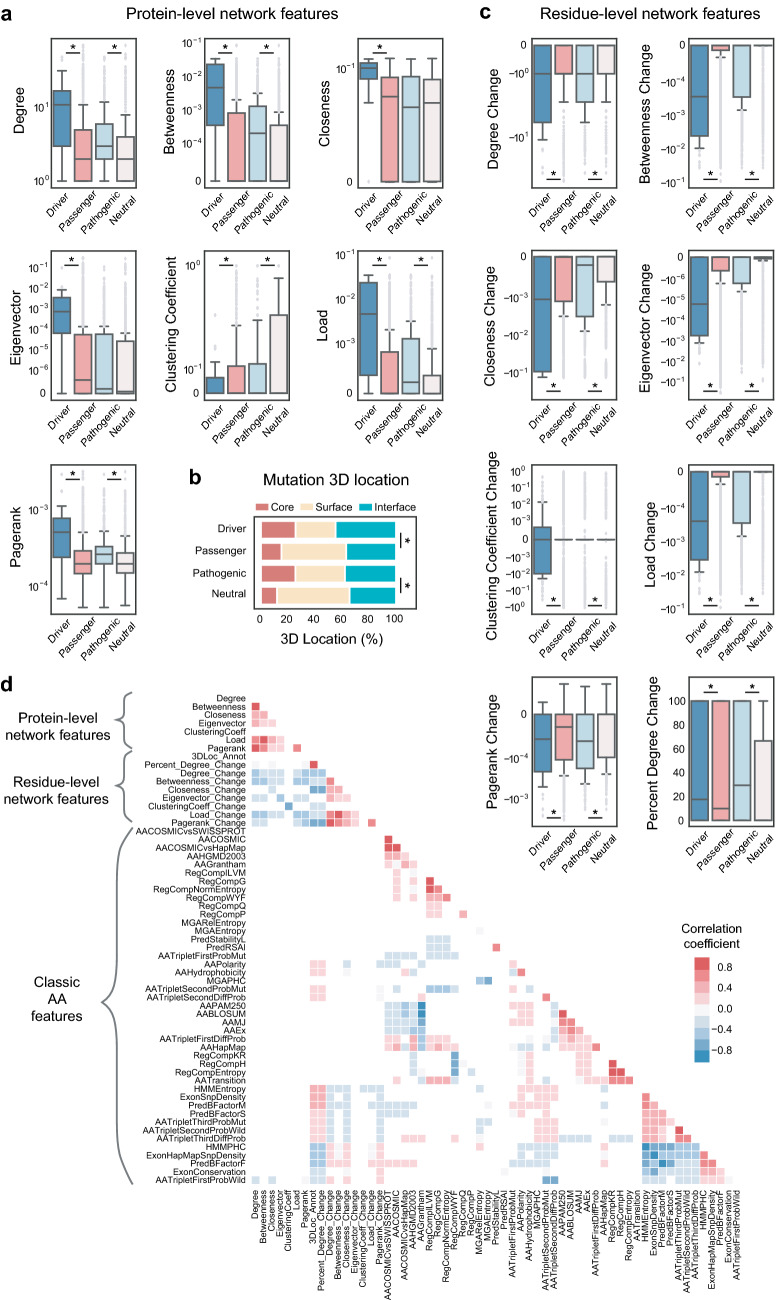
Distribution of network-based features. Distribution of network-based features for driver vs. passenger mutations and pathogenic vs. neutral variants in SRNet (Mann–Whitney *U* test; *p < 1e−04). **a** Boxplots showing distribution of protein-level features (degree, betweenness, closeness, eigenvector, clustering coefficient, load, and pagerank), **b** a stacked bar plot showing percent distribution of 3D locations (core, interface, and surface) of mutations, and **c** boxplots showing distribution of residue-level network features (degree change, betweenness change, closeness change, eigenvector change, clustering coefficient change, load change, pagerank change, and percent degree change). **d** Heatmap displaying Pearson correlation coefficients of network-based and classic non-network-based amino acid features. Only features that have at least one correlation coefficient higher than 0.3 and only values above 0.1 are shown. Classic amino acid features are ordered based on hierarchical clustering of correlation values

As the above analyses support that both protein and amino acid-level information derived from networks is informative about disease-association, we hypothesized that network information would be useful for machine-learning-based variant classification. We designed and analyzed 16 features describing network-level effects of mutations, including 7 protein-level features (Fig. 4a) that estimate the significance of the target protein in the network, and 9 residue-level features (Fig. 4b, c) quantifying the potential of individual amino acid positions on the protein to impact network architecture. The residue-level features are based on comparing network measures before and after removing edges in SRNet potentially affected by a mutation (Materials and methods). These 16 features show potential to distinguish between different classes of variants (Fig. 4a–c) and are not strongly correlated with other classic non-network-based amino acid features used for variant classification, such as measures of site-specific conservation (Fig. 4d), suggesting that they add new and useful information (Fig. S2, Table S2).

### Utility of network features for classifying cancer driver mutations

To evaluate the benefit of using network features for somatic mutation classification, we trained a Random Forest to predict driver or passenger class labels using different combinations of features. We separately evaluated classifier performance when trained using all 16 network-derived features, only the 7 protein-level features or the 9 residue-level features alone, or in combination with 83 amino acid-level features obtained from the SNVBox database (Wong et al. 2011). As a training set, we used likely driver and likely passenger missense mutations from Tokheim et al., which they obtained from TCGA using a semi-supervised approach based on known cancer driver gene annotations and mutation rates with the goal of generating a more balanced training set consisting of both driver and passenger mutations in cancer genes (Tokheim and Karchin 2019). While passengers greatly outnumber drivers in practice, we constrained the ratio as 1:4 driver vs. passenger mutations for classifier training (Materials and methods). Generalization error was estimated using a fivefold cross-validation with gene hold out to prevent information leakage and consequent overfitting. We measured performance using the area under the ROC (auROC) and precision–recall curve (auPRC) metrics, similar to prior variant effect prediction studies (Tokheim and Karchin 2019). We note that use of network features limits training and prediction to mutations that can be annotated by SRNet.

**Fig. 5:**
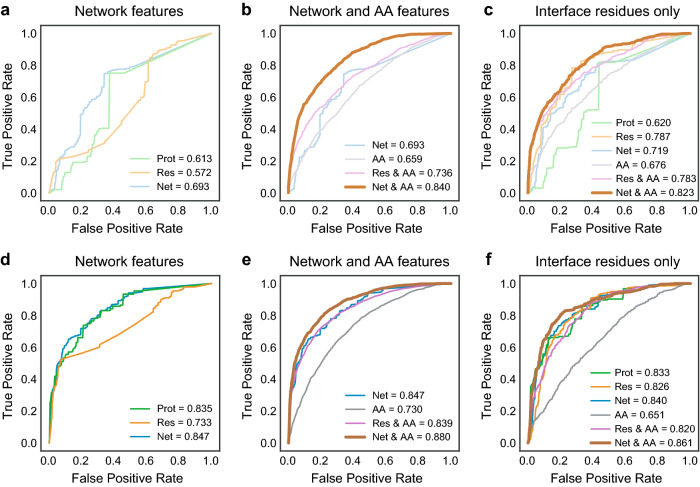
Classifier performance in identifying cancer mutations using SRNet vs. the extended network. ROC curves for identifying cancer mutations using **a–c** SRNet, and **d–f** the extended network, with **a–d** protein-level network features (Prot), residue-level network features (Res), and all network features (Net = Prot + Res); with **b–e** all network features, amino acid features (AA), residue-level network and amino acid features (Res and AA), and all network and amino acid features (Net and AA). **c–f** ROC curves for identifying cancer mutations targeting interface residues only, using all above-mentioned features. ROC curves using Net and AA features are bold. Performance is measured using auROC scores

For driver classification, protein-level network features performed better than residue-specific features (Figs. 5a, S3a), though performance for residue-level features was better for the top scoring ~ 20% of drivers (left edge of ROC curve). We note that residue-level features alone classify all surface non-interface mutations as passengers, since their feature values should all be the same (there is no change to network centrality measures when no edges are affected). Combining residue-level features with more classic amino acid-level features significantly boosts performance over residue-level features alone (Figs. 5b, S3b). Interestingly, network features alone slightly outperform amino acid-level features alone, pointing to the extreme centrality of driver genes. As residue-level features are likely to be most informative for mutations at interfaces, we further explored performance for interface mutations only (Figs. 5c, S3c). Here, we see that residue-specific network features perform considerably better as they are not hindered by misclassification of surface mutations (Figs. 5c, S3c). Overall, the combination of network-based and amino acid features displays the highest performance (Figs. 5b–c, S3b–c). Notably, precision–recall curves indicate that incorporating both network and classic AA features resulted in a significant drop in false-positive predictions relative to either type of feature alone (Fig. S3a–c).

### Incorporating in silico predicted interface residues

The restriction to analysis of mutations for which 3D structural information about interfaces is available is a problematic limitation. In silico prediction of interfaces can be used to augment interface coverage, as done for Interactome INSIDER (Meyer et al. 2018). To explore whether in silico predicted interfaces could boost mutation coverage without loss of performance, we repeated our analysis on an extended network with both structure-derived and predicted interfaces. This resulted in improved performance overall (Figs. 5d–f, S3d–f), suggesting that improvements to interface features and the ability to train on a larger set of mutations enabled by higher coverage in the expanded network outweigh the introduction of noise caused by interface prediction error. We also noted a larger gain in precision for network features relative to AA features when using expanding the network (Fig. S3e). A more stringent comparison considering only proteins shared between the original and the expanded network found similar results (auROC is 0.832 and 0.871 for SRNet and the extended network for the classifier with Network and AA features, respectively).

As we obtained our optimal performance using all features with the extended network, we used this classifier to evaluate feature importances. In Random Forest classifiers, feature importances are determined as the mean decrease in impurity when using that feature to split training examples according to class label during classifier training (Breiman 2001). Fourteen of the top 21 features were network derived, including the top 9 (Fig. S2, Table S2). Protein-level network features were more informative than residue-level features, possibly reflecting the limitation of residue-level features to distinguish surface mutations. Simple 3D location annotating mutations to location at core, surface, or interface contributed less information, which may reflect its redundancy with other network features.

**Fig. 6:**
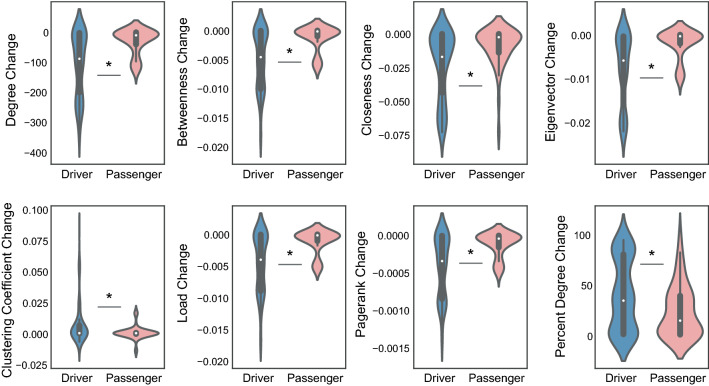
Distribution of residue-level network features. Distribution of residue-level network features for correctly labeled driver and passenger interface mutations occurring in the same proteins (Mann–Whitney *U* test; *p < 0.05)

We further investigated residue-level network features in the extended network by examining cases where the classifier was successful in differentiating between driver and passenger mutations occurring in the same proteins. Since residue-level features only vary within protein for interface mutations, we looked for cancer genes where both driver and passenger mutations at interfaces were correctly classified. We found seven cancer genes (EGFR, HRAS, KRAS, TP53, PIK3R1, CTNNB1, and PTEN) that contained both correctly classified interface driver and interface passenger mutations. Focusing on 212 correctly classified interface mutations in these genes, we observed a significant difference in distribution of residue-level features for the driver and passenger classes (Fig. 6), further supporting that residue-level network features provide information useful for within gene mutation classification.

### Overall performance on benchmark datasets

We next sought to evaluate the improvement obtained from network features on independent studies of cancer mutations. We used our highest performing classifier which is trained on cancer mutations that map to the extended network and all network-based and classic amino acid features (Fig. 5e, Net and AA classifier with auROC = 0.880, Fig. S2, Table S2). Since no ‘gold standard’ dataset exists for cancer, we evaluated classifier performance relative to best-in-class methods that do not use network-derived features on four external pan-cancer datasets constructed using different approaches: an in vivo screen: Kim et al. (2016), an in vitro assay: Ng et al*.* (2018), and two literature-derived datasets: MSK-IMPACT and CGC-recurrent, previously described in Tokheim and Karchin (2019). For each dataset, considering the mutations scored by all methods, classifier performance was evaluated using the area under the ROC (auROC) and PR curves (auPRC), accuracy, F1 score, and the Matthews correlation coefficient (MCC) (Table S3).

**Fig. 7:**
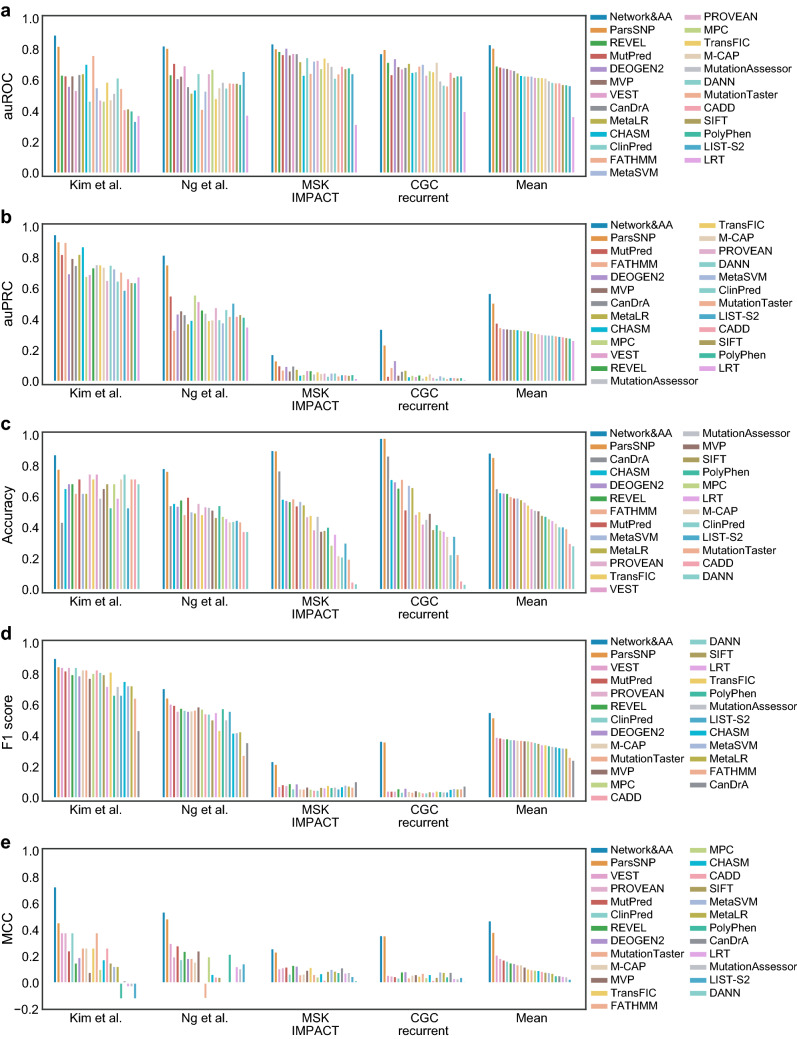
Comparison of classifier performance on benchmark datasets relative to established methods. Bar plots depict **a** the area under the ROC (auROC) and **b** the area under the PR curve (auPRC) scores, **c** accuracy, **d** F1 score, and **e** the Matthews correlation coefficient (MCC) results for each method. Mean category displays the mean of scores of each method across datasets. Methods are ordered based on their mean scores. All panels use the same color scheme

We assessed the performance of our classifier relative to both cancer-specific: CHASM (Carter et al. 2009), ParsSNP (Kumar et al. 2016), TransFIC (Gonzalez-Perez et al. 2012), and CanDrA (Mao et al. 2013), and population-based methods: VEST (Carter et al. 2013), SIFT (P. C. Ng and Henikoff 2003), PolyPhen (Adzhubei et al. 2010), CADD (Kircher et al. 2014), ClinPred (Alirezaie et al. 2018), DANN (Quang et al. 2015), DEOGEN2 (Raimondi et al. 2017), FATHMM (Shihab et al. 2013), LIST-S2 (Malhis et al. 2020), LRT (Chun and Fay 2009), M-CAP (Jagadeesh et al. 2016), MPC (Samocha et al. 2017), MVP (Qi et al. 2021), MetaLR and MetaSVM (Dong et al. 2015), MutPred (Pejaver et al. 2020), MutationAssessor (Reva et al. 2011), MutationTaster (Schwarz et al. 2014), PROVEAN (Choi et al. 2012), and REVEL (Ioannidis et al. 2016) (Fig. 7). Comparison was based on the set of benchmark mutations scored by all methods, and was on the basis of auROC, auPRC, accuracy, F1 score, and Matthew’s correlation coefficient (MCC). We note the later three use discrete labels rather than continuous scores. We used provided labels or recommended cutoffs for all methods as possible, and used a cutoff of 0.5 otherwise (Materials and methods).

Our classifier performed well on all five metrics across the four benchmark sets relative to most other methods (Table S3). It had the highest auPRC (Fig. 7b), F1 score (Fig. 7d), and MCC (Fig. 7e), of all methods on the four benchmark sets, and also performed well on auROC and accuracy (Fig. 7a, c). In most cases, the difference in auROC relative to other methods was deemed significant by the DeLong test (Table S4). After our method, the next best-performing method was ParsSNP, after which there was considerable variation in what methods performed best by various measures. Overall, these results suggest that network-derived features that capture abstract information about the role of proteins in networks and the potential of mutations to perturb this role are helpful for driver classification across a variety of settings, though the gains over methods trained on classic amino acid-based features are modest.

We separately compared our approach to two methods that incorporate interactome related features: SuSPect (Yates et al. 2014) and CHASMplus (Tokheim and Karchin 2019). SuSPect includes protein degree as a predictive feature, while CHASMplus now includes a feature indicating the number of interactions affected if a mutation occurs at a protein interaction interface, along with other improvements relative to the original method. Location at an interface was reported as the second most informative feature after a feature describing within protein clustering of observed mutations (Tokheim and Karchin 2019). We noted improved performance relative to SuSPect and comparable performance to CHASMplus (Fig S4). We note that both our method and CHASMplus derive classic AA features from the SNVbox database (Wong et al. 2011). We further analyzed mutations that were correctly classified by our method but misclassified by SuSPect or CHASMplus to see whether the network features implemented relative to these methods explained the difference. These mutations were significantly enriched at interface regions compared to surface or core (OR 3.78, *p* = 1.45e−11) and they had significantly different distributions of all network features (Mann–Whitney *U* test, *p* < 0.05) apart from closeness change (*p* = 0.22), when compared to mutations misclassified by our method but correctly classified by SuSPect or CHASMplus, suggesting that even though some shared features exist between the methods, our classifier better reflects the network rewiring by mutations.

However, it should also be considered that our network-based approach is dependent on the inclusion of proteins in the network and availability of annotations mapping amino acid residues to core, surface, or interface residues. This generally results in a smaller training set than other methods, and an inability to score some fraction of mutations. For the benchmark sets evaluated here, 95.77% of Kim et al*.*, 72.03% of Ng et al., 78.82% of MSK-IMPACT, and 74.11% of CGC-recurrent dataset mutations could be scored, respectively. It is possible that better network and amino acid annotation coverage could further boost performance.

### Utility of network features for classifying pathogenic germline variants

We next evaluated whether network features are also useful in the context of germline variation. We previously observed that inherited disease genes were less central than cancer genes and both pathogenic and neutral mutations were enriched at interface positions, though to different extents. We once again trained a Random Forest classifier to prioritize missense mutations that alter protein activity using the 16 network-based features and 83 amino acid descriptors, using a training set composed of pathogenic and neutral variants (Materials and methods).

**Fig. 8:**
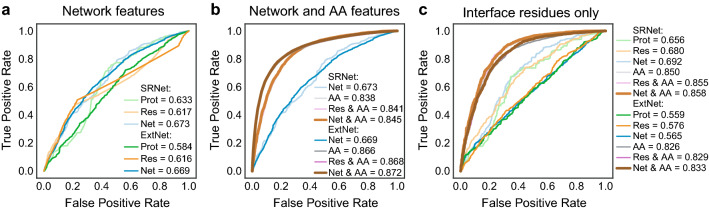
Classifier performances for predicting pathogenic vs. neutral variants using SRNet vs. the extended network (ExtNet). ROC curves for identifying variants with **a** protein-level network features (Prot), residue-level network features (Res), and all network features (Net = Prot + Res); with **b** all network features, amino acid features (AA), residue-level network and amino acid features (Res and AA), and all network and amino acid (Net and AA) features. **c** ROC curves for identifying variants targeting interface residues only using all above-mentioned features. ROC curves using Net and AA features are bold. Performance is measured using auROC scores

For germline variants, residue-specific features yielded similar (with SRNet) or higher performance (with extended network) than protein-level features for all mutations (Figs. 8a, S5a), and for interface mutations only (Figs. 8c, S5c). But overall, network features are outperformed by non-network amino acid features (Figs. 8b, S5b) which is the opposite of the case with cancer driver classifier. This is consistent with proteins targeted by pathogenic germline variants being less central than cancer driver genes. Since proteins harboring germline pathogenic variants have fewer interaction partners, pathogenic variants in the protein core or at interfaces tend not to result in as extreme values of residue-level network features as driver mutations do (Fig. 4c), despite the observed enrichment for these variants in core and interface regions (Fig. 3c–d). The similar performance by network features in both SRNet and the extended network (Fig. 8) suggests that either increased coverage does not improve performance as much, or the noise introduced by interface prediction error counteracts the performance gained by higher coverage in this setting. A stricter comparison considering only shared proteins between networks once again showed similar performance (auROC is 0.835 and 0.849 for SRNet and the extended network for the classifier with Network and AA features, respectively). Precision–recall curves show similar results (Fig. S5).

## Discussion

Understanding the functional consequences of protein coding variants remains a challenging task. Machine learning methods developed thus far to predict whether a mutation is likely to impair protein activity or cause a pathogenic phenotype have largely been protein-centric; however, a growing body of work points to perturbation of the interactome as a major determinant of pathogenicity (Chen et al. 2018; David et al. 2012; David and Sternberg 2015; Engin et al. 2016; Garcia-Alonso et al. 2014; Guo et al. 2013; IMEx Consortium Curators et al. 2019; Kamburov et al. 2015; Nishi et al. 2016; Piñero et al. 2016; Porta-Pardo et al. 2015; Raimondi et al. 2016; Sahni et al. 2013, 2015; Vidal et al. 2011; Wang et al. 2012; Wei et al. 2014; Yates et al. 2014). Such studies of variant distribution in biological systems have provided insights as to how molecular interaction networks evolve to ensure robustness or vulnerability to genetic variation (Capriotti et al. 2019). It is increasingly apparent that the role of proteins within molecular networks is a key determinant of the potential of variants to exert deleterious effects (Chen et al. 2018; Khurana et al. 2013; Yates et al. 2014). Motivated by these studies, we investigate here how network-derived features can capture novel information about variant effects that is not already present in the classical amino acid features used by most variant classification methods, and show that combining both sets of features improves classifier performance.

Our approach relies on a structurally resolved PPI network that allows variants to be characterized according to their potential to affect network architecture by mapping them to their location on protein structures and protein–interaction interfaces. These mappings are used to capture the potential of variant positions to perturb information flow through the network. We developed protein-level features to capture the relative importance of a protein within the network, and residue-level features to capture the potential of mutations to alter the architecture of the network. Though protein-level network features are shared by all variants in a protein, they nonetheless can interact with other amino acid-level features to support classification; in all cases, combining both protein and residue-level network features with classic amino acid features outperformed combining only residue-level network features with classic amino acid features. Residue-level features were helpful for distinguishing between variants within proteins; however, because we designed them to capture the potential of mutations to alter the network architecture, all surface non-interface variants received the same value for these features. We also did not consider the possibility that surface mutations could generate a new edge in the network. Such mutations could be more common in cancer, where missense mutations have been reported to alter binding specificities for kinases and their substrates, thereby remodeling network architectures (Creixell et al. 2015).

Though network features show fairly different distributions for different classes of variants (Fig. 4) and are orthogonal to the features typically used for variant classification, the best classifier combining both feature types shows only modest gains over classifiers that use only classic amino acid features. This may result from more limited availability of training set mutations due to the requirement for structure and interface information to estimate network feature values. This requirement also constrained the coverage of benchmark set mutations that could be classified, though the values remained generally high, and over 70% in the worst case. Performance generally improved when we included predicted protein interactions from Interactome INSIDER (Meyer et al. 2018), suggesting that in silico approaches may be an effective strategy to boost performance until more complete experimentally derived interaction maps are available.

Network features were more informative in the context of somatic mutations than when classifying inherited variants, though performance gains were observed in both cases. This is perhaps expected, since inherited disease genes tended to be less central in PPI networks than cancer genes (Fig. 2), and location at an interface by itself was less discriminatory in the germline setting (Fig. 3d). We speculate that these differences may arise from different selective pressures acting on somatic versus germline variation. Because development at the organismal level is likely dependent on the integrity of molecular interaction networks, both pathogenic and neutral variants may be more constrained by network architecture; whereas in cancer, where selection operates at the cellular level and is predominantly positive, mutations may be better tolerated and be more advantageous in central network positions. We note also, however, that training sets for the driver versus passenger classification problems tend to be more gene-centric leading to concerns over whether cancer mutation classifiers distinguish primarily between genes (Raimondi et al. 2021). Although we made an effort to include both drivers and passengers in each driver gene to mitigate this, it may still be reflected in the higher utility of protein-level network features for driver classification.

In conclusion, our study suggests that information about molecular interaction networks can be incorporated into machine-learning-based variant interpretation frameworks. This opens future directions for the development of novel features capturing network information. Since networks can be constructed to model cell-type and condition specificity (Greene et al. 2015), it may be possible to build classifiers that can capture context-specific effects of variants. Furthermore, as studies have shown that different interfaces are associated with different protein activities, network-based features could make it possible for machine-learning methods to provide more insight into the potential for mutations within a protein to have distinct functional consequences. We anticipate that such advances will boost the utility of variant classification tools for precision medicine applications.

## Materials and methods

Data and code are available at https://github.com/cartercompbio/NetFeatures.

### Source of protein interaction data

To analyze disease gene centrality, we obtained a human PPI network of 12,811 proteins that are involved in 97,376 experimentally verified undirected interactions with a confidence score higher than 0.4 from STRING v11.0 (Szklarczyk et al. 2015).

### Disease genes

A list of 125 high-confidence cancer genes consisting of 54 oncogenes and 71 tumor suppressor genes was obtained from Vogelstein et al. (2013). We also obtained a list of 4524 Mendelian genes from the OMIM database (Amberger et al. 2019). These genes were used to evaluate disease gene centrality (Fig. 2).

### Collecting structural protein and protein interaction data

We obtained human protein interaction data (complete set) from Interactome3D (Mosca et al. 2013), which contains a collection of a highly reliable set of experimentally identified human PPIs. We collected experimental co-crystal 3D structures for 5865 of these interactions from the Protein Data Bank (PDB) (Berman et al. 2003) and homology models for 5768 additional interactions (Mosca et al. 2013) making a total of 11,633 interactions between 6807 proteins with structural protein and interaction data.

### Creating a structurally resolved PPI network

Amino acid residues were annotated as participating in a protein interaction interface based on KFC2 (Zhu and Mitchell 2011) scores, and we removed interactions containing fewer than five interface residues on either partner. Additionally, we calculated relative solvent accessible surface areas (RSA) using NACCESS (Hubbard and Thornton 1993) for all residues in each protein structure. Residues with RSA < 5% and RSA > 15% were designated as core and surface residues, respectively. Residues with RSAs between these thresholds were excluded from further analysis due to ambiguity. When multiple PDB chains were available for the same protein, we used the consensus designation as the final label. The mapping of PDB residue positions onto UniProt residue positions was performed via PDBSWS web server (Martin 2005). After this mapping, we created a structurally resolved PPI network (named SRNet) of 6230 proteins and 10,615 undirected protein–protein interactions with a total of 530,668 interface residues (Table S1). To extend coverage of the structurally resolved network, we defined an extended network based on the “High Confidence” dataset of Interactome INSIDER (Meyer et al. 2018)***, ***a human PPI network of 14,445 proteins with 110,206 undirected interactions containing in silico interface residue predictions in addition to those derived from 3D structures.

### Source of somatic mutation data

To investigate structural location of cancer mutations on proteins (Fig. 3), we mapped more than 1.4 million somatic missense mutations from TCGA (Collins and Barker 2007) onto the structurally resolved PPI network using structural annotations. Only mutations mapping to canonical proteins were used. After this mapping, we identified a total of 56,667 interface residues of 5005 proteins that are involved in 9235 interactions as mutated.

### Training set for cancer mutation prediction

We collected a set of cancer missense mutations designated as likely driver (*n* = 2051) and likely passenger (*n* = 623,992) from Tokheim et al. (Tokheim and Karchin 2019). Of these, 961 driver mutations from 32 genes and 28,043 passenger mutations from 2986 genes mapped to SRNet for a total of 29,004 mutations. All 32 genes with driver mutations also contain passenger mutations. To handle the driver vs. passenger mutation count imbalance in the training set by maintaining an approximate 1:4 driver vs. passenger mutation ratio similar to Carter et al. (2009) while not overrepresenting particular genes, we limited the number of passenger mutations for each gene to 16 (median per gene driver mutation count) and collected 4626 passenger mutations at random across all genes with passenger mutations. In the extended network, we mapped 1513 driver mutations from 52 genes and 118,777 passenger mutations from 4478 genes for a total of 120,290 mutations. Thirty-eight of fifty-two genes with driver mutations also contain passenger mutations. To maintain an approximate 1:4 driver vs. passenger ratio as described above, we limited the number of passenger mutations for each gene to 17 (median per gene driver mutation count) and collected 6549 passenger mutations at random across all genes with passenger mutations.

### Training set for pathogenic variant prediction

We collected 5608 ‘pathogenic’ variants from ClinVar (Landrum et al. 2018), and 3418 neutral variants including ‘common’ variants (allele frequency > 1%) from EXAC (Lek et al. 2016), variants with ‘polymorphism’ classification from SwissVar (Mottaz et al. 2010), and ‘benign’ variants from ClinVar (Landrum et al. 2018), that map to SRNet, totaling 9026 missense variants. We also collected 21,819 pathogenic and 35,522 neutral variants from the same databases that map to the extended network, totaling 57,341 missense variants.

### Features

We designed 16 network-based features to quantify the potential impact of a mutation to the underlying network architecture, comprising 7 protein-level and 9 residue-level features. The 7 protein-level features are degree, betweenness, closeness, eigenvector and load centralities, clustering coefficient, and pagerank of the proteins within the PPI network. They are computed using the NetworkX package of Python and they aim to characterize the centrality of a protein in the network based on measures such as the number of nodes it is directly connected to (degree), the amount of shortest paths it is involved in (betweenness and load), the overall closeness to all other nodes (closeness), its embeddedness (clustering coefficient), and the centrality of its neighbors (eigenvector and pagerank). Nine residue-level features describe mutation 3D location (core, interface, and surface) on the protein and changes in centrality of the protein within the PPI network resulting from mapping the mutation to network edges. Core mutations are assumed to affect all edges in the network, while interface mutations are mapped to corresponding edges in the network, and surface mutations retain all edges. Each interface mutation causes the removal of all edges that they are mapped to. The remaining eight residue-level features are based on this description of how the mutation perturbs the network by capturing degree change, betweenness change, closeness change, eigenvector change, clustering coefficient change, load change, pagerank change, and percent degree change. Non-network-related amino acid-based features (*n* = 83) obtained from the SNVBox database (Wong et al. 2011) describe substitution effects on amino acid biophysical properties, evolutionary conservation of variant sites, local sequence biases, and site-specific functional annotations. Pearson correlation coefficient was used to evaluate feature correlations. Our proposed classifier (used in Fig. 7) uses a total of 99 features consisting of all 16 network-based features (7 protein-level and 9 residue-level features) and 83 non-network-related amino acid-based features. The importance of each feature is computed as the normalized total reduction of the criterion brought by that feature (mean decrease in impurity), also known as the Gini importance (Figure S2, Table S2).

The number of PubMed studies featuring each gene was obtained from the NCBI database (https://ftp.ncbi.nih.gov/gene/DATA/gene2pubmed.gz) for all genes in SRNet with NCBI (Entrez) gene IDs. This was used to assess the potential for protein-level features to be affected by study bias.

### Classifier training

We trained a Random Forest classifier (n_estimators = 1000, max_features = 'sqrt') on the training set using the scikit-learn Python package. To avoid classifier overfitting, we performed prediction using a fivefold gene hold out cross-validation by dividing the training set into 5 random folds for cross-validation while ensuring a balanced number of disease and neutral mutations across the folds. All mutations occurring in the same gene were kept within the same fold. The classifier score represents the percentage of decision trees that classify a mutation as a disease mutation (driver or pathogenic). Receiver Operator Characteristic (ROC) and precision–recall curves were constructed from the classifier scores and the AUC statistic was used as a measure of classifier performance. To compare the performance of different features for identifying disease mutations, we trained different classifiers on different sets of features: all 16 network-based features (Net), dividing network-based features into 7 protein-level features (Prot) and 9 residue-level features (Res), 83 non-network amino acid (AA) features, 83 amino acid features combined with 9 residue-level features (Res and AA), or 83 amino acid features combined with all 16 network features (Net and AA). Training our proposed classifier (used in Fig. 7) on 8062 cancer mutations (1513 driver and 6549 passenger mutations mapping to ExtNet) using all 99 features takes ~ 5.15 s using a Jupyter Notebook on a quad Intel Xeon E5-4650 v4 cpu with a total of 56/112 cores/threads and 512 GB of RAM. Prediction of 100,000 mutations takes ~ 2.27 s.

### Benchmark datasets

We obtained 4 pan-cancer benchmark sets of missense mutations consisting of an in vivo screen: Kim et al. (2016), an in vitro assay: Ng et al. (2018), and 2 literature-derived datasets: MSK-IMPACT and CGC-recurrent from Tokheim and Karchin (2019). The in vivo screen contains 71 mutations selected based on their presence in sequenced human tumors and screened in mice to assess oncogenicity and then labeled as ‘functional’ or ‘neutral’ based on their abundance (Kim et al. 2016). The in vitro assay consists of 747 mutations from a growth factor dependent cell viability assay annotated as ‘activating’ for increased cell viability, or as ‘neutral’ for the remaining, with the assumption that a mutation yielding higher cell viability indicates driverness (Ng et al. 2018). The MSK-IMPACT dataset is composed of mutations from approximately 10,000 tumors (Zehir et al. 2017) on 414 cancer-related genes (MSK-IMPACT gene panel) labeled as positive class if annotated as ‘oncogenic’ or ‘likely oncogenic’ in OncoKB (Chakravarty et al. 2017), or as negative class if not. The CGC-recurrent dataset consists of TCGA mutations annotated as positive class if recurrent in a set of curated likely driver genes from the Cancer Gene Census (Forbes et al. 2017), or as negative class if not.

### Comparison to other methods

Performance was compared to 24 state-of-the-art methods that do not use network-based information, 4 cancer-focused methods: CHASM (Carter et al. 2009), ParsSNP (Kumar et al. 2016), TransFIC (Gonzalez-Perez et al. 2012), and CanDrA (Mao et al. 2013), and 20 population-based methods: VEST (Carter et al. 2013), SIFT (Ng and Henikoff 2003), PolyPhen (Adzhubei et al. 2010), CADD (Kircher et al. 2014), ClinPred (Alirezaie et al. 2018), DANN (Quang et al. 2015), DEOGEN2 (Raimondi et al. 2017), FATHMM (inherited disease version) (Shihab et al. 2013), LIST-S2 (Malhis et al. 2020), LRT (Chun and Fay 2009), M-CAP (Jagadeesh et al. 2016), MPC (Samocha et al. 2017), MVP (Qi et al. 2021), MetaLR and MetaSVM (Dong et al. 2015), MutPred (Pejaver et al. 2020), MutationAssessor (Reva et al. 2011), MutationTaster (Schwarz et al. 2014), PROVEAN (Choi et al. 2012), and REVEL (Ioannidis et al. 2016). We obtained prediction scores for the mutations in the 4 benchmark sets described above for 20 of the methods (VEST, SIFT, PolyPhen, CADD, ClinPred, DANN, DEOGEN2, FATHMM, LIST-S2, LRT, M-CAP, MPC, MVP, MetaLR, MetaSVM, MutPred, MutationAssessor, MutationTaster, PROVEAN, and REVEL) from the dbNSFP database (version 4.1a) (Liu et al. 2020) via the Ensembl Variant Effect Predictor (VEP) (McLaren et al. 2016), and for 4 additional methods (CHASM, ParsSNP, CanDrA, and TransFIC) from Tokheim et al. (Tokheim and Karchin 2019). We also obtained scores on benchmark datasets for two additional methods that use network features: SuSPect (Yates et al. 2014) from (http://www.sbg.bio.ic.ac.uk/suspect) and CHASMplus from Tokheim and Karchin (2019) (Fig. S4).

Classifier performance was compared using the area under the ROC (auROC) and PR curves (auPRC), accuracy, F1 score, and the Matthews correlation coefficient (MCC) (Table S3). Only the mutations scored by all methods were considered for comparison. Significance of difference of auROC measures is evaluated by DeLong test (Table S4). auROC and auPRC values were computed using the predicted scores; while accuracy, F1 score, and MCC were estimated based on the predicted labels (positive vs. negative). For label assignments, we used the provided labels from dbNSFP for SIFT, PolyPhen, ClinPred, DEOGEN2, FATHMM, LIST-S2, LRT, M-CAP, MetaLR, MetaSVM, MutationAssessor, MutationTaster, and PROVEAN. Methods that did not provide labels directly typically provided a score between 0 and 1 (except CADD and MPC), and we ensured that a higher score indicated a more damaging mutation. Where specified we used recommended score cutoffs (0.1 for ParsSNP, 0.7 for MVP, and 50 for SuSPect) for label assignments when evaluating F1 score, accuracy, and MCC results. When no threshold was suggested (or if the suggestion was 0.5), we used a cutoff of 0.5 (our classifier Network&AA, CHASM, VEST, CanDrA, TransFIC, CADD, DANN, MPC, MutPred, REVEL, and CHASMplus). It is important to note that while auROC and auPRC results are independent of the predicted class labels; accuracy, F1 score, and MCC results are dependent on the labels and the threshold used for their assignment; therefore assuming a cutoff of 0.5 could underestimate accuracy, F1, and MCC for some methods.

### Statistical analysis

Distributions are compared using a Mann–Whitney *U* test. Correlations are evaluated using the Pearson correlation coefficient. Odds ratios are calculated using Fisher’s exact test. auROC scores are compared using the DeLong test.

## Supplementary Information

Below is the link to the electronic supplementary material.

Study bias analysis. Correlation of node degree (the number of interacting partners) of proteins in SRNet with the number of PubMed publications they appear in (Pearson r=0.23, p=4.78e-54) (EPS 1512 kb)

Random forest feature importances. The importance of a feature is computed as mean decrease in impurity (MDI), also known as the Gini importance. The network-based features and the amino acid features are colored red and black respectively (EPS 1287 kb)

Classifier performance using precision-recall in identifying cancer mutations using SRNet vs. the extended network. Precision-recall (PR) curves for identifying cancer mutations using **(a-b-c)** SRNet, and **(d-e-f)** the extended network, with **(a-d)** protein-level network features (Prot), residue-level network features (Res), and all network features (Net = Prot + Res); with **(b-e)** all network features, amino acid features (AA), residue-level network and amino acid features (Res & AA), and all network and amino acid features (Net & AA). **(c-f)** PR curves for identifying cancer mutations targeting interface residues only, using all above-mentioned features. PR curves using Net & AA features are bold. Performance is measured using area under the PR curves (EPS 1200 kb)

Comparison of classifier performance on benchmark datasets against existing methods that use network features. Bar plots depict the area under the ROC (auROC) and the area under the PR curve (auPRC) scores, accuracy, F1 score, and the Matthews correlation coefficient (MCC) results for each method. Mean category displays the mean of scores of each method across datasets. All panels use the same color scheme. A SuSpect cutoff of 50 (as recommended in Yates et al. (Yates et al., 2014)), and 0.5 for CHASMplus and the Network&AA classifier were used to assign labels for assessing accuracy, F1 score and MCC (EPS 731 kb)

Classifier performance using precision-recall for predicting pathogenic vs. neutral variants using SRNet vs. the extended network (ExtNet). Precision-recall (PR) curves for identifying variants with **(a)** protein-level network features (Prot), residue-level network features (Res), and all network features (Net = Prot + Res); with **(b)** all network features, amino acid features (AA), residue-level network and amino acid features (Res & AA), and all network and amino acid (Net & AA) features. **(c)** PR curves for identifying variants targeting interface residues only using all above-mentioned features. PR curves using Net & AA features are bold. Performance is measured using area under the PR curves (EPS 1217 kb)

Structurally resolved PPI network SRNet. A structurally resolved PPI network of 6230 proteins and 10,615 protein-protein interactions where 530,668 interface residues located at physical interactions are determined from 3D protein complex structures. Each row describes a residue at an interaction interface in three columns. The first two columns denote UniProt IDs of two proteins (Uniprot1 and Uniprot2) that physically interact. The third column denotes an interface residue of the first protein (Uniprot1_residue) that is in physical contact with one or more residues on the second protein (XLSX 12439 kb)

Features used by our classifier Network&AA ranked based on importance (XLSX 20 kb)

Performances of compared methods on benchmark datasets. For each dataset, considering the mutations scored by all methods, classifier performance was evaluated using the area under the ROC (auROC) and PR curves (auPRC), accuracy, F1 score and the Matthews correlation coefficient (MCC) (XLSX 17 kb)

DeLong test results when comparing auROC measurements of our classifier Network&AA to other methods using benchmark sets. A negative z-score means the method has a lower auROC score than our classifier Network&AA, and a positive z-score means the opposite (XLSX 15 kb)

## Data Availability

The datasets generated and analyzed during the current study are available at https://github.com/cartercompbio/NetFeatures.
