# Publisher Correction: Predicting functional consequences of mutations using molecular interaction network features

**DOI:** 10.1007/s00439-022-02492-3

**Published:** 2022-09-24

**Authors:** Kivilcim Ozturk, Hannah Carter

**Affiliations:** 1 Division of Medical Genetics, Department of Medicine, University of California San Diego, La Jolla, CA USA; 2 Bioinformatics and Systems Biology Program, University of California San Diego, La Jolla, CA USA; 3 Moores Cancer Center, University of California San Diego, La Jolla, CA USA

## Publisher Correction: Human Genetics (2021) 141:1195–1210 10.1007/s00439-021-02329-5

Publisher’s Note: Predicting functional consequences of mutations using molecular interaction network features.

The Publisher regrets that the article by Ozturk, K., Carter, H. (Ozturk and Carter 2022) which is part of this Special Issue: Computational Interpretation of Human Genetic Variation, was published in an earlier issue 141(6):1195–1210.

We would like to express our thanks to the Guest Editors, Prof. Yana Bromberg and Prof. Predrag Radivojac, for their dedication and work assembling this collection of reviews and research for Human Genetics.
